# The Role of Perspective in Mental Time Travel

**DOI:** 10.1155/2016/3052741

**Published:** 2015-12-31

**Authors:** Caterina Ansuini, Andrea Cavallo, Lorenzo Pia, Cristina Becchio

**Affiliations:** ^1^Department of Robotics, Brain and Cognitive Sciences, Istituto Italiano di Tecnologia, 16163 Genoa, Italy; ^2^Department of Psychology, University of Torino, 10123 Turin, Italy; ^3^SpAtial, Motor & Bodily Awareness (SAMBA) Research Group, Department of Psychology, University of Torino, 10123 Turin, Italy

## Abstract

Recent years have seen accumulating evidence for the proposition that people process time by mapping it onto a linear spatial representation and automatically “project” themselves on an imagined* mental time line*. Here, we ask whether people can adopt the temporal perspective of another person when travelling through time. To elucidate similarities and differences between time travelling from one's own perspective or from the perspective of another person, we asked participants to mentally project themselves or someone else (i.e., a coexperimenter) to different time points. Three basic properties of mental time travel were manipulated: temporal location (i.e., where in time the travel originates: past, present, and future), motion direction (either backwards or forwards), and temporal duration (i.e., the distance to travel: one, three, or five years). We found that time travels originating in the present lasted longer in the self- than in the other-perspective. Moreover, for self-perspective, but not for other-perspective, time was differently scaled depending on where in time the travel originated. In contrast, when considering the direction and the duration of time travelling, no dissimilarities between the self- and the other-perspective emerged. These results suggest that self- and other-projection, despite some differences, share important similarities in structure.

## 1. Introduction

When imagining time and their own life events, humans do not only retrieve or predict when events have occurred or will occur, but also automatically “project” themselves on an imagined* mental time line* [1]. Self-time travelling can thus be regarded as the ability to transpose one's habitual self-location in time to different temporal “locations” in the past or the future [2]. Emphasizing the role of perspective taking, this ability to change one's own temporal egocentric perspective has been proposed to share a common mechanism with the ability to change one's own spatial egocentric perspective [3]. In both domains, people would use existing representations as templates for processing and understanding new information, in order to plan their short- and long-term behaviors. On this account, the same processes that subserve simulation of the self at a different location* in space* would also subserve simulation of the self at a different point* in time* [4].

An interesting question, inspired by this parallel, is whether, similarly to taking another person's spatial perspective, people can also adopt the temporal perspective of another person when travelling through time. Studies investigating spatial perspective taking indicate that people can overcome their own position in space to adopt another person's spatial perspective (e.g., [5]). When the scene includes another person, for instance, people may spontaneously describe spatial relations from that person's perspective despite the very real presence of their own [5, 6]. These and other findings suggest that spatial perspective taking may induce an alterocentric remapping, that is, remapping of objects and locations to an alterocentric frame of reference [7].

To our best knowledge, no study has so far investigated whether a similar remapping may take place in the temporal domain. In other words, whether similarly to taking another person's spatial perspective, people can take another person's temporal perspective when travelling through time. To address this issue, in the present study we directly compared self- and other-perspective time travelling with the goal of testing whether/how the representation of time varies as a function of perspective taking.

The psychological ability to traverse temporal distances is dependent upon a cognitive representation of time that has been suggested to be spatial in nature (e.g., [2, 8–12]; for an overview see [13]). In this conceptualization, time travel is characterized by three basic components: (a)* temporal location*, that is, the point in (space-) time from where the travel originates [8, 14, 15]; (b)* motion direction*, that is, the direction along which projection takes place (either backwards or forwards with respect to the temporal location) [8, 14]; and (c)* temporal duration*, that is, the temporal interval to be travelled (for a description of similar concepts, see also [16]). Within this framework, it has been demonstrated that rather than being mapped to space in a uniform manner, spatiotemporal representation presents areas of different granularity. For instance, Christian and colleagues [17] found that when asked to temporally locate events on a time line, participants used more space (-time) to represent one year in the present than in the past or the future. In a similar vein, Arzy and colleagues [14] showed that, irrespective of the temporal location, participants were faster and more accurate when asked to retrieve an event or a face located forwards rather than backwards (relative future effects in [14]; see also [8]). In addition, the speed of self-projection in time has been shown to depend logarithmically on the temporal distance between the imagined self-location in time and the location of the imagined event/face to retrieve [1].

These patterns relate to the self-referenced topography of space-time mapping. To investigate whether similar patterns also apply to other-projection in time, here, we asked participants to imagine themselves or someone else at a specific point in time (i.e., past, present, or future) and “to operate” a notional time machine for travelling either backwards or forwards as to reach a target destination (i.e., one, three, or five years back/ahead). We used the travel duration as a proxy for how space-time representation depended on the perspective taking (self- versus other-perspective). Based on previous evidence that the amount of space used to represent time varies as a function of self-relevance [17], we expected that travelling in the self-perspective would take longer than travelling in the other-perspective. Moreover, we hypothesized that this effect of perspective would be greater when the travel originated in the present than in the past or future. In spite of these differences, however, we also expected similarities between self- and other-perspective time travelling. In particular, we predicted that, for both self- and other-perspective, travel duration would increase as a function of the temporal distance to be travelled. Finally, we expected that, in both perspectives and regardless of temporal location, travelling would be facilitated for the forwards motion direction (see relative future effect [14]).

## 2. Methods

### 2.1. Participants

Twenty-five participants (15 females, aged between 20 and 26 years, mean ± SD 23 ± 1.7 years) from the University of Turin took part in the study. All participants were right-handed and had normal or corrected to normal vision and no history of neurological or psychiatric disorders. All participants gave written informed consent before inclusion in the study, which was conducted in accordance with the principles of the revised Helsinki Declaration [18] and approved by the Ethical Committee of the University of Turin.

### 2.2. Design and Procedure

Participants were seated at a desk approximately 50 cm away from a 17′′ computer screen (refresh rate = 60 Hz). At the beginning of the experiment, they were told that their task was to operate a notional “time machine” to travel through time. Next, they were given instructions about the temporal perspective (self versus other) and the time “locations” at which to imagine themselves. On other-perspective trials, a female coexperimenter was seated at a desk placed perpendicularly with respect to participant's desk (at a distance of ~1.5 m), in front of a computer monitor connected to participant's monitor. While sitting at their desk, participants could not see what was displayed onto the monitor in front of the coexperimenter. To control for possible effects related to age difference, participants were recruited as to be similar in age to the coexperimenter. At the beginning of the experiment, they were informed about coexperimenter's age (i.e., 24 years old).

**Figure 1:**
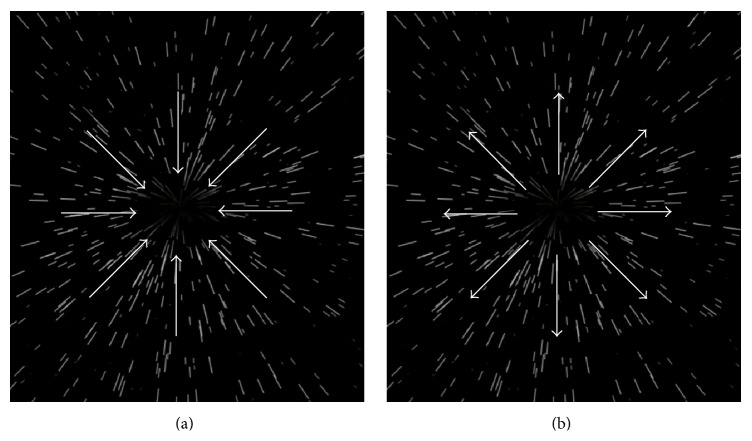
Illustrations of the direction of centripetal (a) and centrifugal (b) optic flow stimuli in trials with backwards and forwards motion direction, respectively.

Each trial began with the instruction to imagine oneself (for self-perspective trials) or the other person (for other-perspective trials) at specific past (i.e., the day of your/coexperimenter's 10th birthday), present (i.e., today), or future location in time (i.e., the day of your/coexperimenter's 50th birthday). As participants were in their late adolescence/early adulthood, past and future locations corresponded to two stages of development markedly distinct from their own (i.e., a point in middle childhood and a point in middle adulthood, resp.). From these locations, participants were instructed to move one, three, or five years, either backwards or forwards. To support the experience of “travelling” through time, an animated star-field display was projected onto the screen in front of the participant [17, 19, 20]. The display consisted of approximately 1000 randomly positioned white dots on a black background (see Figure 1). The dots (i.e., stars) were animated (25 fps) so as to appear to move, on a linear trajectory, either toward (i.e., centripetally) or away from (i.e., centrifugally) the center of the display, corresponding to the experience of backwards and forwards self-motion (Figure 1). Journeys in the past were accompanied by backwards optic flow, while those in future were accompanied by forwards flow (for a similar paradigm see [17]). On other-perspective trials, the same star-field display was projected onto the screen in front of participant and onto the screen in front of the coexperimenter. In both self- and other-perspective trials, the participant was instructed to look at the screen in front of him/her for the entire duration of the experiment. At a self-paced interval, participants were requested to press a button (i.e., space bar) to begin the time travel (i.e., initiate the optic flow) and press it again once they felt they had reached the target (i.e., stop the optic flow). The “time travel” duration was calculated as the interval between these two events. Finally, to ensure participants' compliance with the task requests, in 20% of the experimental trials (a total of 72 trials per each participant, with 18 trials per each block), we asked participants to estimate the actual duration of their last “time travel” (in ms).

Self- and other-perspective trials were administered in different blocks. The order of presentation was either ABBA or BAAB. Each of the four blocks comprised 90 trials for a total of 360 trials. For each perspective condition (self, other), ten trials for each temporal location (past, present, and future) by motion direction (backwards, forwards) by temporal duration (one, three, and five years) combination were administered. Within each block, temporal location presentation was fully randomized and administered in miniblock of 6 trials (i.e., one trial for each temporal duration by motion direction combination). To familiarize participants with the procedure, at the beginning of the experiment a practice session was administered (6 self-perspective and 6 other-perspective-trials, one trial for each motion direction by temporal duration combination).

E-Prime V2.0 software (Psychology Software Tools Inc., Pittsburgh, PA, USA) running on a PC was used to present trials and record the duration of the “time travel” (i.e., the time elapsed between starting and stopping the “time machine”). The experiment lasted about 60 minutes.

### 2.3. Statistical Analyses

To cope with the high variability within the range of travel duration (394–21304 ms), we converted individual temporal intervals data into *z*-scores, based on means and standard deviations computed over all trials per each participant. Since each standardized coefficient scales appropriately to adjust for the disparity in the variable sizes, this procedure makes it possible to bring all of the variables into proportion with one another without losing the possibility to directly compare participants' performance across conditions. The mean *z*-scores were then averaged separately for each trial type (i.e., temporal location by each motion direction by temporal duration in both perspectives). The investigation of standardized data distribution using one-sample* Kolmogorov-Smirnov goodness-of-fit* tests did not show any significant difference, suggesting that data distribution within the sample was Gaussian (0.196 < *p*_*s*_ > 0.998). Travel duration *z*-scores were then submitted to a repeated measures ANOVA with* temporal location* (past, present, and future),* motion direction* (backwards, forwards),* temporal duration *(1, 3, and 5 years), and* perspective* (self, other) as within subjects factors. Main effects were used to explore the means of interest (post hoc *t*-test), and Bonferroni's corrections (*α* level of *p* < 0.05) were applied.

In addition, to obtain an indirect measure of participants' compliance with the task requests over the experimental session, we analyzed the data from the control task to test whether participants' ability to reproduce time intervals decreased/increased over the four blocks. To this aim, we first computed a* temporal accuracy estimation index*, defined as the difference between the actual duration of the travel and the corresponding temporal estimation given by the participant at the control task trial (i.e., 20% of the total amount of trials: 18 trials per each block for a total of 72 trials by each participant). Then, we submitted this index to a repeated measures ANOVA with* block* (i.e., first, second, third, and fourth) as within subjects factor.

## 3. Results

**Figure 2:**
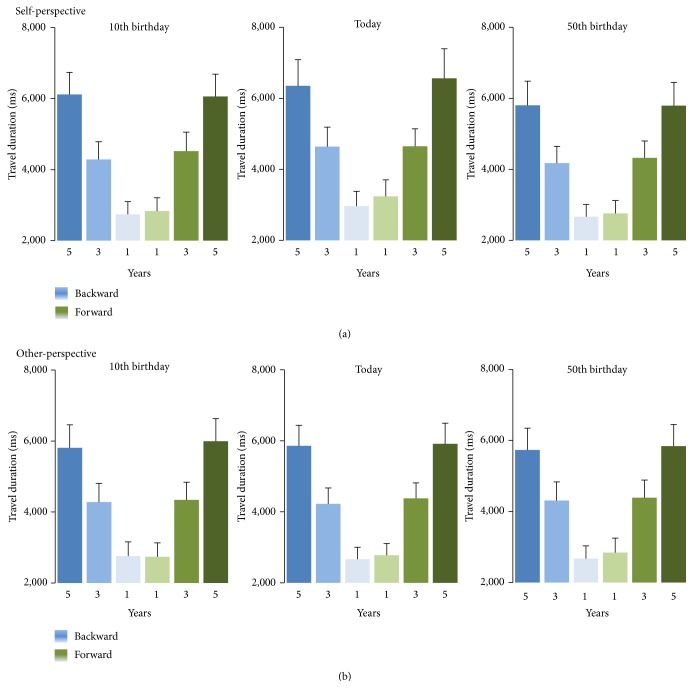
Durations (ms) for time travelling one, three, or five years backwards (blue tone bars) or forwards (green tone bars) from the past (10th birthday), the present (i.e., today), or the future (i.e., 50th birthday) in self-perspective (a) and other-perspective (b). Error bars depict standard error of mean.

*Mental Travel Task.* Participants' travel duration varied as a function of temporal location so that travels originating in the present moment were longer than travels originating in the past or in the future (main effect of* temporal location*: *F*_(2,48)_ = 3.621; *p* = 0.034; partial eta square = 0.131; mean *z*-scores = 0.078,  0.004, and −0.082, resp.; *p*_*s*_ < 0.05). Moreover, temporal duration of journeys was longer for travelling forwards than backwards (main effect of* motion direction*: *F*_(1,24)_ = 7.305; *p* = 0.012; partial eta square = 0.233; mean *z*-scores = 0.038 versus −0.038, resp.; *p*_*s*_ < 0.05). Regardless of the temporal origin of the journey and its direction, the travel duration increased as a function of the number of years to travel (main effect of* temporal duration*: *F*_(2,48)_ = 277.747; *p* < 0.001; partial eta square = 0.920; mean *z* scores = −0.86 for 1 year; −0.01 for 3 years; 0.86 for 5 years; see Figure 2, *p*_*s*_ < 0.05). As predicted, neither* motion direction *nor* temporal duration* main effect were further qualified by significant interactions by* perspective*. In contrast, the effect of* temporal location* varied between self- and other-perspective (*perspective* by* temporal location* interaction: *F*_(2,48)_ = 6.014; *p* = 0.005; partial eta square = 0.200). For self-perspective trials, indeed, travel duration was longer when the travel started either in the past or in the present rather than in the future (0.034 and 0.163 versus −0.101, resp.; see Figure 2; *p*_*s*_ < 0.05). For other-perspective trials, in contrast, no similar modulation of travel duration by temporal location was reported (−0.007, −0.025, and −0.062 for present, past, and future location; Figure 2; *p*_*s*_ > 0.05). Moreover, travel duration was on average longer for the self-perspective than for the other-perspective when the travel started in the present, but not when it started in the past or in the future (self/present versus other/present = 0.163 versus −0.007, resp.; *p* < 0.05; self/past versus other/past = 0.034 versus −0.025 and self/future versus other/future = −0.101 versus −0.062, resp.; *p*_*s*_ > 0.05). Finally, the exploration of the significant* temporal location* by* temporal duration* interaction (*F*_(4,96)_ = 3.780; *p* = 0.007; partial eta square = 0.136) revealed that there was no difference across temporal locations when travels lasted either 1 year or 3 years (1 year: past = −0.897; present = −0.793; future = −0.912 and 3 years: past = −0.016; present = 0.057; future = −0.075; *p*_*s*_ > 0.05). In contrast, when participants were requested to cover a 5-year distance, the trip took longer when it started in the past or present rather than in the future (5 years: past = 0.926 and present = 0.968 versus future = 0.74; *p*_*s*_ < 0.5). Neither the main effect of* perspective* (*F*_(1,24)_ = 1.588; *p* > 0.05; partial eta square = 0.062) nor the remaining two-, three-, or four-way interactions were found to be significant (all *F*_*s*_ < 1.484;  0.213 < *p*_*s*_ > 0.942; all partial eta squares < 0.058).

*Control Task. *ANOVA revealed no significant effect of* block* (*F*_3,72_ = 2.212; *p* > 0.05; partial eta square = 0.084) on temporal accuracy estimation index, suggesting that participants' compliance to the task request remained stable throughout the experiment.

## 4. Discussion

In this study, we assessed whether and how self- and other-projections in time map onto similar or different spatiotemporal representations. For the properties of this underlying representation to emerge, we used as a proxy the time taken to move through time (i.e., travel duration) and manipulated three basic properties of time journeys: temporal location (past, present, or future), motion direction (backwards or forwards), and temporal duration (one, three, or five years).

Our results suggest that self- and other-projections hinge on different temporal representations depending on the temporal location, that is, on where in time the mental travel originates. Specifically, for self-perspective, participants took longer to cover identical distances when the travel started in the past or in present compared as to when it started in the future. For other-perspective, in contrast, travel duration was not modulated by temporal location. This effect may reflect the tendency to form higher-level construals of information about remote future event [21]. In this respect, Trope and Liberman [21] suggest that “the greater the temporal distance from a future event, the more likely is the event to be represented abstractly in terms of a few general features that convey the perceived essence of events rather than in terms of more concrete and incidental details of the event.” In the same vein, D'Argembeau and Van der Linden [22] provided evidence that projecting oneself in a specific positive or negative experience results in a richer representation when the event is expected to be experienced in the near future rather than in a more distant future. On this account, participants would travel more rapidly from a remote future location because they would simulate future events in more abstract and general terms. This is further supported by the consideration that, in contrast with remembering of past events whose features are already integrated, simulating remote future events requires the combination of disparate details gleaned from a variety of episodic sources [23]. Forming abstract representations of remote future event may thus serve a specific adaptive role in reducing the costs required to integrate unrelated details into a coherent future representation.

Over and above this, self- and other-projection also scaled differently for mental travels taking place in the proximity of the present moment. For the present location, regardless of motion direction, one year was indeed longer in the self-time rather than in the other-time. This was not the case for one-year travels originating from a past or future location. This pattern is in agreement with the finding that participants represent self-time as occupying a greater amount of space than an equivalent period related to others [17]. Of direct relevance to the present study, Christian and colleagues [17] asked three different groups of participants to “time-travel” from the present moment to their own birthdays or to the birthdays of either a close friend or a hypothetical stranger of a similar age as theirs. It was found that, irrespective of motion direction (either in the past or in the future), time relevant to self was represented as occupying more space than time relevant to others (i.e., best friend or unfamiliar other). On a closer examination, however, this effect of self-relevance was only evident for temporally close events (i.e., 10 years before or after the present moment), but not more distant events (i.e., the day of 8th or 58th birthday). Taken together with our results, this suggests that a distinctive relationship bounds the “self” to the “now.” As observed by Núñez and Cooperrider [16], indeed, within an “internal” perspective, the ego is always and inherently colocated within the “now.” It is perhaps therefore not surprising that self-projection is more “embedded” in the representation of the present time compared to other-projection. On a related note, this could explain why recent autobiographical memories tend to be recalled from the first-person perspective, while more remote memories, particularly early childhood memories, are more likely to be recalled from a third-person perspective [24–27].

Despite these differences, however, self- and other-projections also shared many similarities in structure. First, for both self- and other-perspectives, travel durations increased as a function of temporal duration (one year < three years < five years). With only one exception (i.e., five years from past and present lasts longer than five years from future), this effect was not modulated by the temporal location (i.e., past, present, and future). This is consistent with the idea that computation of temporal quantities rests on a common system for magnitude processing [28], which is (at least in part) independent of self-relevant content specificity and episodic memory processes. The finding that 5-year travel duration lasted less when it originated in remote future might be taken to suggest that, as far as remote future is concerned, the spatial representation of time is compressed towards the anchoring point. Experiments using multiple temporal locations in remote past and future might help to address this issue.

Second, in both self- and other-perspectives, independent of temporal location, travelling forwards took longer than travelling backwards. The neural system subserving mental time travel has been proposed to have evolved to anticipate and pilot our behavior rather than primarily encoding the past [8, 29, 30]. In line with this, processing of events has been shown to be future oriented across present, past, and future self-location [8, 14]. For example, when asked to judge whether an event takes place before (relative past) or after (relative future) an imagined self-location in time, participants are typically faster and more accurate for relative future than for relative past events [8].

At first sight, the finding that travelling forwards took longer than travelling backwards may seem contradictory to response facilitation for future events. However, both phenomena may be parsimoniously interpreted as resulting from an anisometric pattern of internal spatial representation of relative past and future events, such that the representational medium is compressed towards the relative past and dilated towards the relative future. Compression towards the relative past would explain why travelling backwards lasts less, but also why judgements are more difficult (i.e., slower and less accurate) to the relative past. Along the same lines, dilation towards the relative future may account for both the increase in travel duration for travelling forwards and the relative future response facilitation.

An alternative, not mutually exclusive, explanation of the motion direction effect refers to discrepancy between external time direction (i.e., roughly, the time of the calendar) and subjective time direction (i.e., the personal time which is measured by the traveler's wristwatch; [31]) potentially experienced when travelling backwards. When participants are requested to time-travel towards relative future, the external time and their subjective time move towards the same direction (see also [32], for similar concepts). The subjective time may therefore be expected to add to the external time, extending the travel duration. In contrast, when participants travel towards relative past, the external time goes backwards, while their subjective time moves forwards [32]. The time traveler may therefore experience a shrinking of travel duration. On this account, the effect of motion direction would reflect the relative (forward) motion of the traveler making journeys end later in the future and earlier in the past. At this stage, both these hypotheses remain speculative and require further study for elaboration and validation.

## 5. Conclusions

A growing body of evidence indicates that self-projection in space and in time might rest on a common neural network and share similar cognitive processes and representations [3]. In the visuospatial domain, it has been documented that people can transpose their own actual point of view and “navigate” space from the perspective of someone else [5]. The current findings suggest that a similar ability might also exist in temporal domain, supporting the notion of temporal perspective taking.

By contrasting time travelling from self- and other-perspective, we found evidence that temporal representation underlying one's own projection shares many of the same characteristics of the temporal representation underlying another person's projection. Despite the fact that a greater “sensitivity” to temporal location for representing time in self- rather than in other-perspective emerges, when considering more abstract properties as direction and magnitude, self- and other-time exhibit a similar structure. Further research is warranted to clarify whether and to what extent these effects are sensitive to the degree of similarity between the self and the other person. For example, it will be important for future research to determine how travelling in time from the perspective of a younger or older person impacts on travel duration (depending, e.g., on whether the past/future of the participant overlaps with the present of the other person).

A second issue to be addressed in future studies relates to the neural underpinnings of self- and other-projection. Self-projection in time has shown to recruit a network of brain areas in distinct time periods including the occipitotemporal, temporoparietal, and anteromedial temporal cortices [4, 8]. For example, it has been reported that during mental time travel the left lateral parietal cortex is differentially activated by nonpresent subjective times compared with present (past and future > present) [33]. Capitalizing on the finding that left parietal cortex supports first-person perspective simulation [34], these results have been interpreted to suggest that the parietal cortex is specifically related to transformations in subjective time. Moreover it has been demonstrated that temporal self-projection into the personal past recruits greater ventral medial prefrontal cortex (mPCF) whereas self-projection into another person's perspective recruits greater dorsal mPCF [35]. Asking participants to simulate mentally past, present, and future time from their own versus another person's perspective might help to clarify how transformations in subjective and nonsubjective time are represented in left parietal cortex and to elucidate the exact contribution of the ventral and dorsal subregions of mPCF to self- versus other-projection. Finally, the hypothesis of a partial overlap between self- and other-mechanisms for projection in time could be tested in neuropsychiatric patients with temporal orientation failures [36]. To the extent that self- and other-projection rely on a common neural mechanism, self-referenced and other-referenced disorientation may be expected to share common fundamental characteristics.
